# An Approach to Identifying Headache Patients That Require Neuroimaging

**DOI:** 10.3389/fpubh.2019.00052

**Published:** 2019-03-15

**Authors:** Andrew Micieli, William Kingston

**Affiliations:** ^1^Division of Neurology, Department of Medicine, University of Toronto, Toronto, ON, Canada; ^2^Department of Neurology, Woman's College Hospital, Toronto, ON, Canada

**Keywords:** neuroimaging, headache, neurology, migraine, MRI

## Abstract

Headache is one of the most common clinical scenarios faced by a neurologist or neurologist in training. However, the decision process on when to complete neuroimaging can be difficult in clinical practice. This article focuses on a well-organized and evidence-based approach to identify patients with headache that require neuroimaging and will lend confidence to the clinician faced with these scenarios in clinical practice. The approach includes neuroimaging in episodic migraine, chronic migraine, identifying secondary headache disorders in clinic and the emergency department, and discusses pitfalls to over imaging. The article concludes with a flowchart to summarize an overall clinical approach.

## Introduction

A framework for when to complete neuroimaging in patients with headache is an important skillset for a neurologist, neurologist in training, emergency physician, internist, or general practitioner. Simply, when is it appropriate to order neuroimaging? Does the patient with chronic migraine require a magnetic resonance imaging (MRI) study? What imaging does a patient with a thunderclap headache require in the emergency department? When is vascular imaging indicated? What are characteristics of neurological symptoms to suggest it is secondary to a focal cerebral lesion as opposed to a migraine aura?

The reality of clinical practice is patients are often over imaged for fear of missing uncommon but important intracranial pathology. Physicians may also feel compelled or pressured to complete imaging, when the likelihood of finding intracranial pathology may be the same as the general population. There are potential risks to the patient and society with this approach in a resource-restricted health care system.

**Figure 1:**
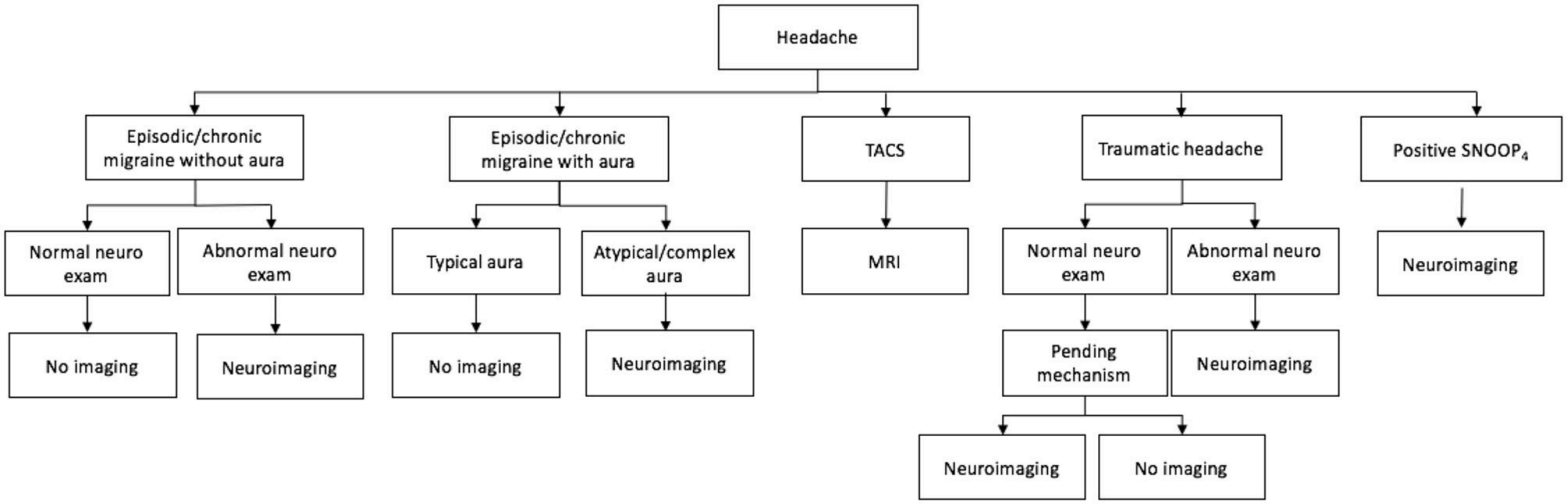
Approach to neuroimaging in a patient with headaches. TACS, trigeminal autonomic cephalalgias.

There are no available studies that allow for definitive recommendations on neuroimaging in headache patients, however this article will focus on an evidence-based and well-organized approach and will lend confidence to the physician faced with these common scenarios in clinical practice. The authors created a flowchart (see Figure 1) that may be helpful as a general clinical approach. It can be applied to a broad patient demographic including patients seen in clinic, the emergency department, and the inpatient hospital ward.

## Neuroimaging in Migraine

Primary headache (i.e., migraine and tension headache) are the majority of headache patients presenting to a primary care practice, 76% of which are migraine (1). Migraine is the third most prevalent disorder worldwide and second most disabling, affecting more women than men (2). According to the *International Classification of Headache Disorders, 3rd Edition* (ICHD-III) criteria, migraine attacks should last between 4 and 72 h, and have at least two of the four following criteria: (1) unilateral location, (2) pulsating pain, (3) moderate to severe intensity, and (4) aggravated by routine physical activity (3). There must also have at least one of the following: (1) nausea and/or vomiting and (2) photophobia and phonophobia.

Approximately 0.1% of headaches are sinister (i.e., secondary headaches, which include neoplasm, aneurysm rupture, venous sinus thrombosis, meningitis, etc.) (4). Among patients with migraine and a normal neurological examination, the prevalence of significant intracranial abnormalities on neuroimaging ranges from 0 to 3.1% and combining this data in a meta-analysis resulted in a prevalence of 0.18% (4). Specifically, the prevalence of arteriovenous malformations is 0.8% and saccular aneurysms is 2.4% on autopsy. Although there are many causes of secondary headache, clinical cues to their diagnosis will be present on history and neurological examination which will be discussed below.

In 1994, the American Academy of Neurology (AAN) created a guideline for the use of neuroimaging in patients with headaches and a normal neurological examination, which has not changed over the years (5). The AAN consensus concluded “in adult patients with recurrent headaches that have been defined as migraine, including those with visual aura, with no recent change in pattern, no history of seizures, and no other focal neurological signs or symptoms, the routine use of neuroimaging is not warranted.” The most common abnormalities found on MRI in migraineurs are white matter lesions localized in the subcortical or periventricular white matter best seen on fluid-attenuated inversion recovery (FLAIR) images, reported in 12–48% of migraineurs compared with 2–11% of control subjects (6). These lesions are non-specific and can be misinterpreted as signifying an inflammatory disease, such as multiple sclerosis, leading to patient anxiety, and further investigations, such as a lumbar puncture (with its associated risks). If atypical headache features are present and the patient does not meet ICHD-III criteria for migraine, a lower threshold for neuroimaging may be applied.

Visual aura may precede the migraine, or may not be followed by a headache in the case of migraine aura without headache. Diagnostic features of a visual aura include features such as propagation of a scintillating scotoma, zig-zag lines, fortification spectra, or photopsia. These positive visual symptoms may be a result of a migraine, but very rarely could be attributable to a focal seizure or occipital lobe ischemia (7). There are key clinical features of positive visual phenomenon that warrant neuroimaging to rule out an occipital lobe lesion (such as a cavernoma or AVM) (8). These include: a stereotypical visual aura that is repeatedly experienced in one hemifield, an increase in frequency or change in pattern of a longstanding visual aura, a sudden alteration in aura characteristics, any unexplained visual field defect and/or subjective persistence of a scotoma following a typical visual aura, or co-existence of seizures. If any of the above criteria are met, then neuroimaging with an MRI is indicated.

## A Case of Transformation from Episodic to Chronic Migraine

A 35-year-old female with a history of rheumatoid arthritis and episodic migraine without aura, treated with ibuprofen and Sumatriptan presents to family medicine clinic with increased frequency of migraine episodes over the last 6 months. In the last few months they are also occurring upon wakening in the morning. Her migraines are now approximately 20 days per month and she is using Sumatriptan more than usual. Her rheumatoid arthritis is active and she is using Ibuprofen almost nightly for pain relief. Her baseline migraine frequency is 4–6 days per month. She is referred to the Headache clinic with a documented normal examination and a recently completed brain CT scan that is unremarkable. Did this patient require a CT scan? Do they now require a brain MRI?

To make a diagnosis of chronic migraine a patient needs to have headaches for ≥15 days per month (for >3 months) with migraine features present for ≥8 days (3). Transformation from episodic to chronic migraine occurs in ~3% of migraine patients per year (9). Analgesia overuse is a major risk factor for this transformation. Patients who overuse analgesia are at risk for medication overuse headache (MOH) where the analgesia leads to the paradoxical effect of increasing headache frequency. This is likely the cause of increased migraine frequency in the above example. Maximum monthly use of a non-steroidal anti-inflammatory is 15 days per month, which was exceeded by the patient in the above case (3). Maximum monthly use of a Triptan is 10 days per month. Treatment of MOH is beyond the scope of this paper, however recognition of this phenomenon is important as this patient did not require neuroimaging with a clinical history consistent with a known primary headache disorder, MOH and a normal documented neurological examination and absence of red flags.

In a study of 373 patients with chronic headache referred to a tertiary referral center for increased severity of symptoms or resistance to appropriate drug therapy, change in characteristics or pattern of headache, or family history of an intracranial structural lesion, only 1% (4 scans) showed significant lesions- two osteomas, one low grade glioma, and one aneurysm (10). Of these patients, only the aneurysm was treated. If a patient with headache presents to a primary care clinic with increased frequency of headache (rather than a change in headache characteristics), then a detailed history should be performed to investigate for possible internal or external precipitating factors, such as increased stress/anxiety, sleep deprivation, dietary changes (changes in diet, increased caffeine intake, or caffeine withdrawal), compliance with headache preventive medications, medication overuse or head trauma.

## Clues on History for a Secondary Headache Disorder

Every headache history/exam should attempt to elicit red flags or worrisome clinical features that may signify the presence of an underlying pathological condition requiring neuroimaging. A commonly used published acronym is SNOOP_4_ (see Table 1) (11). If these features are addressed, the chance of overlooking a sinister cause for headache are greatly diminished. MRI is preferred over CT scan, however in the acute setting, especially in the emergency department a CT scan could be performed first, depending on the patient's symptoms. MRI is more sensitive, particularly for lesions in the posterior fossa, as well for neoplasms, cervicomedullary lesions, pituitary lesions, intracranial hyper/hypotension, and vascular disease (arterial and venous infarctions) (12).

**Table 1:** Commonly used acronym to that may signify the presence of an underlying pathological condition requiring neuroimaging.

	**Stands for**	**Example**	**Differential diagnosis**
S	**S**ystemic symptoms	Fever, weight loss, fatigue	Infection (meningitis, encephalitis), giant cell arteritis, metastases, leptomeningeal carcinamatous
	**S**econdary risk factors	Malignancy, immunosuppression, HIV	
N	**N**eurologic symptoms/signs	Focal neurologic deficits, altered consciousness, confusion	Mass lesion, stroke, hydrocephalus
O	**O**nset	Thunderclap, abrupt	Most common include: subarachnoid hemorrhage, reversible cerebral vasoconstriction syndrome, pituitary apoplexy, cerebral venous sinus thrombosis, vasculitis
O	**O**lder (especially >50 years)	New onset, progressive headache	Mass lesion, giant cell arteritis
P	**P**ositional	Change lying vs. sitting	Intracranial hypotension
	**P**rior	Different in quality from baseline	Mass lesion
	**P**apilledema	Visual obscurations	Idiopathic intracranial hypertension
	**P**recipitated by	Valsalva, coughing, sneezing	Posterior fossa lesion

Firstly, onset of maximum pain is important. A thunderclap headache (“worst headache of my life”), by definition reaches its maximum intensity within 1 min or less. It has an associated differential diagnosis, of which the most worrisome is bleeding into the subarachnoid space from a ruptured cerebral aneurysm (13). An urgent plain CT head should be performed looking for blood in the subarachnoid space, and if negative a lumbar puncture is indicated. However, these investigations are insufficient for a patient presenting with a thunderclap headache to the emergency department. Although it is sensitive enough to exclude a subarachnoid hemorrhage, vascular imaging with a CT angiogram +/– a CT venogram is warranted to investigate for other possible thunderclap headache etiologies such as reversible cerebral vasoconstriction syndrome (RCVS), or other less likely possibilities including vasculitis, cervical artery dissection, or cerebral venous thrombosis. CT angiogram is more sensitive than MR angiogram (12). Vascular imaging should be done in the case of a thunderclap headache, a family history of aneurysms or headaches that are continuously ipsilateral or progressive in nature. New onset headaches or change in headache characteristic/pattern in patients on anticoagulation warrants an MRI, specifically with gradient-echo sequence that is sensitive for hemosiderin and calcification, to assess for cerebral microhemorrhages.

An abnormal finding on neurological examination triples the odds of finding a significant intracranial abnormality on neuroimaging, although the odds are still low (i.e., <3 in 100) (4). A history of headache worsening with valsalva maneuver significantly increased the odds of findings a significant intracranial abnormality on neuroimaging, particularly a Chiari malformation.

Intracranial hemorrhage, meningitis, and cerebral neoplasm rarely present with headache as their sole presenting symptom (12). Instead they may present with other focal neurological deficits, fever, laboratory signs of infection, known primary malignancy or constitutional symptoms that suggests a secondary cause of headache. These patients should be initially investigated with a CT head and then MRI if necessary. Identifying secondary headaches in patients over the age of 50 is clinically challenging. Importantly, only ~2% of migraineurs have their first headache over the age of 50 (12). In patients over the age of 65 who present to neurologists with new-onset headache up to 15% may have serious pathology such as stroke, giant cell arteritis, neoplasm, or subdural hematoma (14).

Cerebral venous sinus thrombosis (CVST) must be considered in the right clinical context. This includes, patients with hematologic prothrombotic states (i.e., antithrombin III deficiency, protein C or S deficiency, antiphospholipid antibody syndrome etc.), malignancy (especially hematological malignancy) pregnancy and puerperium, high risk medications (oral contraceptive pill), infections, and systemic diseases (lupus, inflammatory bowel disease, Bechet disease, etc.) (15). The headache in CVST is most often secondary to increased intracranial pressure secondary to impaired venous drainage. Headache may be the only symptom, so vigilance and clinical suspicion is important. CVST may present as a thunderclap headache, a progressive headache over days or weeks despite conservative management, or as an atypical headache. A plain CT scan of the head will miss many cases. Only one third of CVST demonstrates direct signs on CT head (i.e., hyperdense vessel or delta sign) (15). Venous imaging (CT venogram or MR venogram) must be ordered to assess the venous system. Invasive imaging with cerebral angiography should be reserved for cases where CT venogram or MR venogram is inconclusive or when an endovascular procedure is being considered.

Headache is the most common presenting symptoms of idiopathic intracranial hypertension (IIH) or also known as pseudotumor cerebri, however the headache is variable and non-specific (16). Some patients describe headache exacerbation with changes in posture. The often refractory nature of the headache is a clinical cue, in the right patient demographic (i.e., overweight women of childbearing age). Tinnitus and transient visual obscurations lasting seconds often accompany the headache, occurring in two-thirds of patients with papilledema (16). An MRI brain with gadolinium and MR venogram should be ordered to rule out other potential intracranial pathologies, meningeal process or venous thrombus and secondly looking for radiographic signs of raised intracranial pressure (empty or partially empty sella, prominent subarachnoid space around the optic nerves, vertical tortuosity of the optic nerves, intraocular protrusion of the optic nerve head, venous sinus stenosis, and slit-like ventricles) (17). Contrary, low-pressure headache, or headache caused by reduced intracranial cerebral spinal fluid pressure (i.e., intracranial hypotension) can be caused by trauma (even trivial trauma), lumbar puncture, craniotomy, or spontaneously in patients with connective-tissue disorders. In some cases, spontaneous intracranial hypotension may be entirely cryptogenic. The headaches of intracranial hypotension are positional (relieved by lying down). MRI brain findings most often seen include brain descent, caudal displacement of the tonsils, diffuse pachymeningeal enhancement, and bilateral subdural fluid collections (12). MRI brain and spine with contrast should be ordered, and at times identification of the leak is seen.

Giant cell arteritis (GCA) is an inflammatory disease of medium and large vessels. The most feared complication is irreversible vision loss with involvement of the fellow eye if not recognized quickly. Although most clinical manifestations are non-specific, headache is the most common symptom, occurring in more than two-thirds of patients (18). The headache has no specific defining characteristics. An erythrocyte sedimentation rate (ESR) and c-reactive protein (CRP) should both be ordered in these patients, since ESR may be normal in up to 16.6% of biopsy-proven GCA patients (19). An MRI can be used to investigate the mural thickness, contrast enhancement and lumen diameter of the temporal artery (20).

Patients with trigeminal autonomic cephalalgias (TACS), characterized by unilateral head pain associated with prominent ipsilateral cranial autonomic features (lacrimation, conjunctival injection, rhinorrhea) require an MRI brain once at initial presentation to exclude intracranial pathologies accounting for their “side-locked” headache. Pituitary and peri-pituitary gland pathology can present phenotypically as a TACS (21). Examples of TACS include cluster headache, paroxysmal hemicrania, short-lasting unilateral neuralgiform headache attacks with conjunctival injection and tearing (SUNCT), short-lasting unilateral neuralgiform headache attacks with cranial autonomic symptoms (SUNA), and hemicrania continua (3). Any “side-locked” headache (i.e., headache always on the same side) should be imaged once, preferably with an MRI, to exclude an ipsilateral intracranial lesion.

Headache is often the most common sequelae from head injuries (22). For most individuals a traumatic headache gradually dissipates over several days, weeks, or months. In the acute setting, any focal neurological symptoms or signs immediately following a head injury should be promptly evaluated with a CT head and CT angiogram of the head and neck vessels to assess for the presence of a subdural or epidural hematoma, carotid or vertebral artery dissection, cerebrospinal fluid leak or rarely CVST or carotid-cavernous fistula (22). Bone windows should be included with the plain CT head to assess for fractures at the vault or base of the skull. In the absence of findings on the neurological examination, different imaging rules or guidelines have been adopted such as the CT head rules (23). In the chronic post-traumatic setting (weeks, months, or years) for patients with persistent headache with or without postconcussive symptoms, no diagnostic evaluation guidelines exist. MRI is more sensitive and should include a gradient weighted sequence to identify the presence of hemosiderin deposition. Other neuroimaging technologies such as diffusion tensor imaging, magnetic transfer imaging, magnetic source imaging, magnetic resonance spectroscopy, and functional magnetic resonance imaging are currently being investigated (22).

## When to Order Neuroimaging with Contrast

Gadolinium-containing contrast agents are often used in MRI to enhance the quality of images (10). Care is needed in patients with impaired renal function to avoid the rare, but serious adverse effect of nephrogenic systemic fibrosis. Headache patients that require MRI with gadolinium include: patients with abnormal neurologic examination, positional headaches, exertional or valsalva maneuver-exacerbated headaches, cluster or neuralgia-type headaches or facial pain, and known history of cancer, AIDS, immunocompromised, or infectious disease (12).

## Pitfalls to Over Imaging

“Therapeutic scans” may be appreciated by patients and their families, however it can complicate and confuse the situation. One of the difficulties with ordering MRIs in headache patients is the relatively high frequency of “false positive studies” or incidental findings, and in inexperienced hands can be misinterpreted for the cause of the patient's headache. Often they are not of any clinical significance to the patient's headaches or relevant clinically and can worry the patient, or require serial imaging. False positive studies include normal anatomic variants, transverse sinus asymmetry, non-specific white matter lesions, developmental venous anomaly, lipoma, prominent perivascular spaces (Virchow-Robin spaces), cysts, arachnoid granulations, small meningioma, or pituitary adenomas (12). They may lead to further investigations with a lumbar puncture and its associated risks. Other risks to over imaging include false reassurance from an inadequate study, the rare risk of an allergic reaction to iodine contrast media with CT scanning, radiation from CT scans, and the risk of over-sedation in claustrophobic patients having MRI scans.

Older studies have looked at the cost-effectiveness of completing brain imaging in headache patients. In one study, 592 neurologically intact patients were examined between 1990 and 1993 for the complaint of headache. No patient was found to have serious intracranial pathology detected by CT scan (24). The societal cost implications are significant.

## Conclusion

When to order neuroimaging can be a challenging decision faced by the clinician taking care of a patient with headaches. It is further complicated by incurred costs to the health care system and the potential medical-legal consequences. There are no available studies that allow for definitive recommendations on neuroimaging in headache patients. We do however have reasonable evidence-based studies and prevalence estimates, for example in patients with migraine and a normal neurological examination and significant intracranial pathology. We created a framework to help the clinician with an organized approach to neuroimaging in headache patients, and can be tailored on a case-by-case basis.

## Author Contributions

AM conceived of manuscript, involved in writing, and editing the manuscript. WK involved in writing and editing the manuscript.

### Conflict of Interest Statement

The authors declare that the research was conducted in the absence of any commercial or financial relationships that could be construed as a potential conflict of interest.
